# Comment on suspicious for malignancy cytology: nuclear characteristics deserve special attention in reported cytology analysis - real-world scenario cohort in thyroidology

**DOI:** 10.20945/2359-4292-2026-0002

**Published:** 2026-01-28

**Authors:** Ilker Sengul, Demet Sengul

**Affiliations:** 1 Thyroidology Unit, Giresun University Faculty of Medicine, Giresun, Turkey; 2 Division of Endocrine Surgery, Giresun University Faculty of Medicine, Giresun, Turkey; 3 Department of General Surgery, Giresun University Faculty of Medicine, Giresun, Turkey; 4 Department of Pathology, Giresun University Faculty of Medicine, Giresun, Turkey

Dear Editor,

We write in response to the recently published manuscript, “In suspicious for malignancy thyroid nodule aspirates, nuclei characteristics deserve special attention in reported cytology analysis - real world scenario cohort”, published in volume 69, Arch Endocrinol Metab ^(1)^. This diligent retrospective review of 289 nodules with suspicious for malignancy (SFM) cytology, though present findings offer important findings for those involved in thyroid cytopathology, certain aspects invite further discussion. The dedication to identifying diagnostic criteria within the SFM category, especially those that may serve in order of filtering out the rare instances of false positivity, is truly commendable in thyroidology. The most striking revelation of this valued Brazilian study is that the presence of micronucleoli and/or irregular or oval nuclei was the characteristic most strongly associated with malignancy. Indeed, the presence of micronucleoli alone was highly predictive, as shown by de Macedo and colleagues ^(1)^, increasing the chance of malignancy nearly thirteenfold.

This finding suggests that these features, when present, may warrant classifying a specimen as malignant rather than merely SFM. Interestingly, features traditionally emphasized in the literature - such as nuclear grooves and pseudoinclusions, which appeared with great frequency in the reports - did not significantly predict malignancy in this particular cohort. Notably, these established criteria were present in specimens proven to be malignant, as well as in those histologically confirmed as benign, thereby rendering them statistically irrelevant in differentiating true malignancy in this series. Nevertheless, while the reported risk of malignancy (ROM) of 98% in this Brazilian cohort surpasses the 74% mean ROM cited by the latest Bethesda System for Reporting Thyroid Cytopathology (TBSRTC), one must consider the methodology carefully ^(1)^.

The authors stated their outcome of category I, TBSRTC, was 20%; howbeit, would that or other possible desinence(s) alter if finer ones had been utilized? ^(2-7)^ The provider performing the fine-needle aspiration biopsies was a referral specialist of long standing, whose skill may perchance have introduced a selection bias, favoring the high ROM calculated. Howbeit, the analysis was conducted merely upon the cytopathological reports reviewed, rather than a direct examination of the slides themselves. Even though the authors demonstrate that the qualitative nature of the analysis still yielded valuable insights, it limits the ability to perform quantitative assessments that might truly clarify which set of criteria, and how many, must minimally be required to avoid diagnostic failure. Nonetheless, this rigorous analysis underscores the diagnostic importance of nuclear features - especially micronucleoli and nuclear irregularity - for practitioners aiming to maintain a high positive predictive value within the SFM category of TBSRTC. This issue merits further investigation. We thank de Macedo and cols. ^(1)^ for their high-toned research in SM cytology in Arch Endocrinol Metab, for thyroidologists.

## Data Availability

datasets related to this article will be available upon request to the corresponding author.
